# Gene expression in large pedigrees: analytic approaches

**DOI:** 10.1186/s12863-015-0311-z

**Published:** 2016-02-03

**Authors:** Rita M. Cantor, Heather J. Cordell

**Affiliations:** Department of Human Genetics, David Geffen School of Medicine at UCLA, 695 Charles E. Young Dr, South, Los Angeles, CA 90024-7088 USA; Institute of Genetic Medicine, Newcastle University, International Centre for Life, Central Parkway, Newcastle upon Tyne, NE1 3BZ UK

## Abstract

**Background:**

We currently have the ability to quantify transcript abundance of messenger RNA (mRNA), genome-wide, using microarray technologies. Analyzing genotype, phenotype and expression data from 20 pedigrees, the members of our Genetic Analysis Workshop (GAW) 19 gene expression group published 9 papers, tackling some timely and important problems and questions. To study the complexity and interrelationships of genetics and gene expression, we used established statistical tools, developed newer statistical tools, and developed and applied extensions to these tools.

**Methods:**

To study gene expression correlations in the pedigree members (without incorporating genotype or trait data into the analysis), 2 papers used principal components analysis, weighted gene coexpression network analysis, meta-analyses, gene enrichment analyses, and linear mixed models. To explore the relationship between genetics and gene expression, 2 papers studied expression quantitative trait locus allelic heterogeneity through conditional association analyses, and epistasis through interaction analyses. A third paper assessed the feasibility of applying allele-specific binding to filter potential regulatory single-nucleotide polymorphisms (SNPs). Analytic approaches included linear mixed models based on measured genotypes in pedigrees, permutation tests, and covariance kernels. To incorporate both genotype and phenotype data with gene expression, 4 groups employed linear mixed models, nonparametric weighted U statistics, structural equation modeling, Bayesian unified frameworks, and multiple regression.

**Results and discussion:**

Regarding the analysis of pedigree data, we found that gene expression is familial, indicating that at least 1 factor for pedigree membership or multiple factors for the degree of relationship should be included in analyses, and we developed a method to adjust for familiality prior to conducting weighted co-expression gene network analysis. For SNP association and conditional analyses, we found FaST-LMM (Factored Spectrally Transformed Linear Mixed Model) and SOLAR-MGA (Sequential Oligogenic Linkage Analysis Routines –Major Gene Analysis) have similar type 1 and type 2 errors and can be used almost interchangeably. To improve the power and precision of association tests, prior knowledge of DNase-I hypersensitivity sites or other relevant biological annotations can be incorporated into the analyses. On a biological level, eQTL (expression quantitative trait loci) are genetically complex, exhibiting both allelic heterogeneity and epistasis. Including both genotype and phenotype data together with measurements of gene expression was found to be generally advantageous in terms of generating improved levels of significance and in providing more interpretable biological models.

**Conclusions:**

Pedigrees can be used to conduct analyses of and enhance gene expression studies.

## Background

Genome-wide expression studies are making significant contributions to the identification of risk genes for complex traits. Expression studies can help identify genes in linked and associated regions that are appropriate for follow-up with functional studies [1]. Most often knowledge of gene expression in a relevant tissue can help. That is, if a chromosomal region is linked to a phenotype such as one relating to an eye disorder, genes that are expressed in the eye become the prime candidates for further study. In addition, genes that are overexpressed in the eyes of affected individuals compared to controls are also excellent candidates. Also, those genes that fall within the same biological network as a candidate gene are good candidates for further study. There are also studies where gene expression is the prime genetic data (that is, no markers have been genotyped for linkage or association studies). Expression is assessed genome-wide to identify patterns of expression. In all of these studies, we usually do not include biological relatives, so that all observations are independent. The Genetic Analysis Workshop (GAW) 19 data, however, provides expression in multiple members of large pedigrees, giving an excellent opportunity to learn about the familiality of expression and develop methods to adjust for it or capitalize on it in statistical analyses.

### Early microarray analyses

Genetic epidemiologists currently have the ability to successfully quantify transcript abundance of messenger RNA (mRNA), genome-wide, using microarray technologies [2]. For a given gene, and among all genes, mRNA abundance is quite variable, with substantial differences among individuals, tissues, and time periods over a life span [3]. The wealth of data generated over multiple tissues and time points by the recently developed technologies permits investigators to design and conduct studies that promise to substantially improve our understanding of factors that influence mRNA levels. This should then, among other goals, lead to the identification of the elements responsible for their regulation. We anticipate that this growing insight and information will ultimately lead to more precise predictions of gene expression levels by revealing the genetic contributors to regulation, and by providing clarification of how genetic factors act through gene expression to contribute to protein levels and human phenotypes. That is, identifying the ways in which transcript variation is regulated and quantifying the interrelationships of mRNA abundance among genes is expected to help us understand how gene expression contributes to variation in complex human traits. The development of this information is likely to involve a long and intense process, and we are currently in its early stages. However, a great deal of experimentation and analytic work has already been done, and it will facilitate the accuracy, speed and breadth of analyses that contribute to this overarching research aim.

The gene expression group of 9 GAW19 papers tackled some timely and important questions that should contribute to this aim by using established tools developed by others, developing newer tools, and developing extensions to these tools. In addition to this work, the papers we summarize here also evaluated the type 1 and type 2 errors of analytic methods used, provided analytic tools for the research community, and conducted analyses to better understand biological aspects of gene expression. Here we only present a summary of the papers that is designed to place the GAW19 gene expression studies within the context of this broad and evolving field. We hope to help the reader interpret GAW19 investigations and their results. Because this paper is a summary, we encourage those who are interested to read the individual GAW19 gene expression papers for relevant details and to assess the motivations for these works. To place the GAW19 gene expression papers within a larger context, we begin by summarizing some of the methods developed prior to GAW19 that have been applied to gene expression levels, historically, and follow this with a more in-depth presentation of some of the analytic methods used in the GAW19 papers from this group. We then provide a discussion of how the findings reported in these papers can impact the field.

Analytic methods for research using expression data that are derived from microarray technologies have been under development since the inception of these technologies in the early 2000s and during the period of their refinement, which continues to be an ongoing process. The arrays have almost always been used on samples of independent individuals, so that most of the methods developed prior to those by our GAW19 gene expression group did not address capitalizing on the possibilities of large numbers of nonindependent samples, such as those from pedigrees. An exception occurs with early studies of expression quantitative trait loci (eQTL) that used pedigree linkage analyses to map regulatory elements, although most of the later investigations used association studies with single nucleotide polymorphism (SNP) arrays involving samples of independent individuals. eQTL studies are addressed in greater detail below.

Several of the initial statistical methods we mention for gene expression analyses were already available and obvious choices, and others were developed or adapted specifically to address gene expression questions. A salient feature is that expression arrays allow us to query expression levels across the entire genome, simultaneously, giving a much broader view than was previously feasible. Unfortunately, along with this ability, multiple testing, which is tied to the number of probes measured in each study sample, becomes a challenge. For GAW19, approximately 22,000 probes were used; more current array data would include expression measures from 450,000 probes.

### Identifying gene expression differences using false discovery rates

Initially, because of cost, the sample sizes of the studies conducted were relatively small, sometimes as small as 30 individuals, and the extensive multiple testing made statistical power a prohibitive problem. However, the false discovery rate (which is less stringent than a family-wise error rate addressed by a Bonferroni correction), was subsequently applied to analyses of the genome-wide expression data. The false discovery rate (FDR) is set in advance by the investigator to allow for a particular proportion of false positives within the reported positive results. For example, the FDR may be as permissive of 0.05, which would allow 5 % of the tests reported as positive to be incorrect. This statistical criterion was formally described by Benjamini and Hochberg [4], was applied to gene expression studies by Storey and Tibshirani [5], and was the first alternative to the family-wise error rate to gain broad acceptance.

### Cleaning microarray data

In addition to introducing a fundamental difference in the statistical approach to estimation of errors, there was a significant focus on identifying the best methods to clean the array-based expression data. Cleaning involved the identification and removal of outliers resulting from systematic errors in the application of arrays, such as placing cases on separate arrays from controls, and individual errors resulting from the poor preparation of DNA. Storey first described expression heterogeneity in his surrogate variable analysis paper [6], referencing the importance of identifying the sources of batch effects. The cleaning methods are now well developed, although one must always be cognizant of where possible bias could be introduced, and address whether there are factors leading to batch effects in generating expression levels that could affect the results of a study.

### Comparing gene expression in different contexts

Early array-based gene expression studies primarily compared expression levels within different contexts, such as the presence or absence of a disease or the presence or absence of a treatment to cells from individuals in the same disease state [7]. This work is done in case–control studies. T-tests, analyses of variance, and nonparametric versions of these tests were used to identify significant differences in the expression of genes between the two states. Significant differences in gene expression could then be used to identify genes and pathways that are involved in the disease state or the response to treatment [8].

### Finding clusters of genes with similar expression patterns in different states

Early analytic approaches also included methods to cluster genes based on similarities or correlations in their expression levels. The goal was to reveal similarities or differences in coordinated gene expression under different states. Clustering allowed the subdivision of the whole set of genes based on which ones were expressed at higher levels and which ones at lower levels. These similarities are likely to reflect similarities in gene function. For example in a person who has an infection, cluster analyses of gene expression are likely to illustrate that expression of certain immune response genes are elevated in a similar fashion. One could apply a treatment and observe whether the changes in gene expression revealed anything about the biology of the infectious agent or the response to treatment. That is, clustering applied established analytic techniques to the quantitative gene expression levels so as to provide an assessment of similarity in these gene expression levels and cluster the genes according to this similarity. A set of genes that are all highly expressed when compared to the other genes on the array will be in the same cluster and this fact might derive from the impact of a similar genetic or environmental factor or both. This approach clearly answers a different question than asking whether the genes are differentially expressed within differing contexts. This is also different from clustering subjects based on their gene expression levels, which is what is done in evolutionary studies to find similarity among species.

The 2 standard approaches used to find genes that are related through similarity in expression levels are hierarchical and K-means clustering [9, 10]. Key questions should be addressed prior to conducting cluster analyses. These include whether to analyze all genes measured on the arrays or only a selection, as there are genes that will add noise to the analysis because they do not contribute to the clusters. A prior understanding of the biological process under analysis can help identify the genes to select. In addition, nonindependence of replicate samples can lead to biased results and individual study designs may have to be developed to achieve a sufficient sample size such that the results will not be vulnerable to this factor. This is particularly true in pedigree data, which is a key feature of the GAW19 expression data.

Hierarchical clustering lends itself to an easily interpreted visual display of a dendrogram, where the individuals in the study are used to generate the gene expression data and the analyses are conducted to cluster genes with similar expression patterns; the clusters generated by this method, however, are fairly imprecise, such that small changes in expression levels can result in dendrograms that are different. There are 2 methodological approaches. The first method, agglomerative hierarchical clustering, is a “bottom up” approach, where each observation starts in its own cluster, and pairs of clusters are merged as one moves up the hierarchy based on applying an algorithm to the gene expression levels. The second method, divisive hierarchical clustering, uses a “top down” approach, where all observations start within 1 cluster, and the clusters are split recursively as one travels down the developing hierarchy. An alternative approach, K-means clustering [11], requires the investigator to set the number of clusters into which the genes will fall in advance. One begins with an initial partition and the results undergo iterations until a final criterion is reached.

### Weighted gene coexpression network analysis: reducing the dimensionality of gene expression data

As time passed, and more expression data were generated, opportunities to develop novel analytic approaches presented themselves and the gene expression microarray technology matured. More sophisticated analytic methods were developed. Weighted gene coexpression network analysis (WGCNA) is an example of one such widely used method that was employed by a number of the individuals in our GAW 19 group. The method is designed to construct gene networks from the pairwise correlations of expression data [12]. WGCNA allows for the incorporation of context differences and trait values with the gene expression summary measures. WGCNA is presented in much greater detail in the “Methods” section below. More recently, other molecular biology approaches to measure gene expression have become available. RNA sequence data allow for the integration of expression and genotype information measured simultaneously. However, the GAW19 data were array based.

### Identifying genetic contributors to gene regulation: expression quantitative trait loci

eQTL have been studied extensively [13]. Their identification is essential to the search for genetic contributors to gene regulation [14]. eQTL are based on quantified gene expression that can be viewed like any other phenotypic trait and genetic markers can be used for gene mapping through linkage and/or association. The key difference is that there are many such traits generated by microarrays for many genes throughout the genome. Analytically, each of these traits is analyzed the same way as other quantitative phenotypes such as height and weight. Thus, expression traits can be adjusted for covariates and transformed to achieve a normal distribution for quantitative trait loci (QTL) analysis. The key difference here is that a substantial correction for multiple testing is needed to identify eQTL, as the expression levels are usually all tested for linkage or association with all of the SNPs available in the same study sample, engendering a large multiple testing problem, the magnitude of which is the product of the number of gene probes and the number of SNPs. eQTL, whether discovered by linkage or association, identify loci that harbor genetic elements that regulate the expression of the gene under analysis. Those that are next to the gene tested (usually between 50,000 base pairs and 1 megabase, depending upon the preference of the investigator) are classified as *cis* loci, whereas those anywhere else in the genome are classified as *trans* loci [15].

The GAW19 data provided by the workshop organizers included gene expression levels measured on the individuals from 20 pedigrees that were ascertained for individuals with type 2 diabetes. Additional traits included both simulated and real longitudinal measures of systolic (SBP) and diastolic blood pressure (DBP) and whole genome sequence data that has been imputed within the 20 pedigrees. The data are described in detail in the accompanying summary publication [16]. A prior manuscript analyzing a larger sample of the data derived from the San Antonio Family Heart Study reports that 85 % of lymphocyte expression levels were significantly heritable, making them appropriate candidate traits for eQTL analyses in the GAW19 pedigrees. In that manuscript [17], heritability varied substantially among the transcript levels, and the median was 22.5 %. In the published analysis, eQTL were identified by mapping the transcript levels using the SOLAR (Sequential Oligogenic Linkage Analysis Routines) software [18] to conduct linkage analyses.

### GAW19 gene expression group analyses

The 9 papers contributed to GAW19 by our gene expression group explored 3 aspects of gene expression. The first group of papers considered the expression values in the pedigree members without incorporating genotype or trait data into the analysis. The questions explored involved identifying aspects of the correlation structure of the expression levels of the thousands of genes measured. Analytic approaches to accomplish this included principal components analysis [19, 20], WGCNA [20], meta-analyses [20], gene enrichment analyses [20], and linear mixed models [19, 20].

The second group of papers explored the genetics of gene expression by incorporating SNPs and rare-variant genotypes into the gene expression analyses to better allow us to identify contributors to gene regulation. Factors addressed included eQTL complexity [21], the feasibility of applying allele-specific binding (ASB) to filter potential regulatory SNPs [22], and epistatic interactions of eQTL [23]. Analytic approaches to conduct these investigations included linear mixed models [21], measured genotypes in pedigrees [21], permutation tests [23], and covariance kernels [22].

The third group of papers incorporated both genotype and phenotype data into the gene expression analyses to understand the effects of gene expression and/or genetic variation on phenotypic traits. Genome-wide gene expression was used (a) to predict blood pressure phenotypes via its associations with the SNP genotypes [24], (b) to predict hypertension [25], (c) in the joint analysis of blood pressure traits with sequence data [26], and (d) to identify causal models that include blood pressure traits and genotypes with the expression levels. Analytic methods employed in this work included linear mixed models [24, 27], nonparametric weighted U statistics [26], structural equation modeling [27], Bayesian unified frameworks [27], and multiple regression [25].

## Methods

### Analytic approaches

Several well-established analytic approaches have been applied by members of the GAW19 gene expression group to perform analyses involving the gene expression data. We describe the ones that are most applicable to our full set of gene expression analyses in detail here and present refinements and specific applications of them in the Results and Discussion section. The first method, WGCNA, is a recently developed approach that identifies biologically plausible patterns of gene expression from the correlations among genome-wide gene expression data. The second, linear mixed models, is used to test SNP and trait associations with expression levels, while adjusting for the statistical nonindependence of members of the same pedigree. The third, causal modeling, allows for the integration of gene expression, genotype, and trait data in a single analysis for model selection. Here we provide a summary of these analytic approaches.

### Weighted gene coexpression network analysis

WGCNA [12] is a data-mining method that is used to analyze pairwise correlations among gene expression levels to identify networks among genes. The result of the correlation analyses is a set of coexpression gene modules, intramodular hubs, and network nodes. An important aspect of this work is that it reduces the dimensionality of the data, so that multiple testing becomes less of an issue than if each gene expression probe is analyzed separately. It also provides insights into which genes may be operating in the same pathways and the biological processes of the individual genes. To use this method, one first defines a gene coexpression similarity measure, which is used to construct the network. The coexpression similarity measure for a pair of genes, *i* and *j*, is denoted by: *S*_*ij*_. Many studies use the absolute value of the correlation as an unsigned coexpression similarity measure,

$$ {S}_{ij}^{unsigned}=\left|cor\left({x}_i,{x}_j\right)\right|, $$

where gene expression profiles *x*_*i*_ and *x*_*j*_ are the gene expression levels of *i* and *j* across multiple samples. The absolute value does not allow one to discriminate between gene repression and activation. Signed networks allow the similarity between genes to discriminate among these differences. To define a signed coexpression measure between gene expression profiles *x*_*i*_ and *x*_*j*_, a transformation of the correlation,

$$ {S}_{ij}^{unsigned}=0.5+0.5cor\left({x}_i,{x}_j\right) $$

is used. An adjacency matrix (network) is used to quantify how strongly the genes are related to each other. A module eigengene, the first principal component of the standardized expression profiles, can be considered as a summary of the standardized module expression data. To find modules that relate to a clinical trait of interest, module eigengenes are each correlated with the clinical trait of interest, which give rise to eigengene significance measures, allowing one to identify the correlated clinical traits, thus relating gene expression profiles to other phenotypes.

### Linear mixed models

Linear models have a wide application in genetics studies. A linear model expresses the expected value of a trait as a linear combination of observed predictors. A linear model can have fixed, random, or a combination of fixed and random effects predictors. Fixed and random effects refer, respectively, to population-average and subject-specific effects, where the latter are generally assumed to be unknown for an individual. These random effects are usually assumed to have a normal distribution. A linear mixed model is a linear model that contains both fixed and random effects, and thus is composed of mixed effects. These models are very useful when measurements are made on groups of related subjects, for example, in pedigrees. For the eQTL analysis we used the linear mixed effects models where the genetic effects are modeled as random. Using the subscript *kl* to denote the *l*^th^ individual in the *k*^th^ family, and defining *Y*_*kl*_ and *SNP*_*kl*_ as the gene expression and genotype dosage, respectively, we write the model as:

$$ {Y}_{kl}={\beta}_o+{\beta}_{s\ }SN{P}_{kl}+{\alpha}_{kl}+{\varepsilon}_{kl} $$

where the betas denote the regression coefficients for the fixed effects, *α*_*kl*_ is the random intercept, and *ε*_*kl*_ is the normally distributed error term with mean 0 and variance *σ*_*ε*_^2^. The *α*_*kl*_ within the *k*^th^ family are normally distributed with mean 0 and covariance matrix: 2*σ*_*g*_^2^*ϕ*, where *ϕ* is the kinship matrix; the overall covariance matrix is block diagonal with 1 block per family.

The FaST-LMM (Factored Spectrally Transformed Linear Mixed Models) software [28] uses a linear mixed modeling approach to test SNP association with quantitative traits (such as expression levels) in pedigrees. FaST-LMM is designed to perform genome-wide association studies (GWAS) when the relationships among the individuals in the study sample are unknown. Carefully chosen GWAS SNPs genotyped on the study sample are used to estimate genetic similarity. Linear mixed models capture these relationships and transformation of the estimated matrix of pairwise relationships is used to speed the analysis.

### Causal modeling

Structural equation modeling (SEM) is a regression-based approach to causal modeling, popular in the social sciences literature. A system of linear equations is constructed based on hypothesized relationships between variables (nodes) in a graphical model. The resulting structural equation model implies a particular structure for the covariance matrix of the measured variables. Given the observed (empirical) sample covariance matrix, the parameters of the model can be estimated using maximum likelihood. Once the model’s parameters have been estimated, the resulting model-implied covariance matrix can be compared to the observed covariance matrix to assess whether the model provides a good fit to the data, or two different models can be compared with one another using the Akaike information criterion (AIC).

An alternative, recently proposed approach that can be used for modeling multivariate phenotypes is the Bayesian unified framework (BUF) [29]. Although not originally designed for causal modeling per se, the approach allows phenotypic “outcome” variables (including measures of gene expression) to be partitioned into subsets *γ* = (*U, D, I*) with respect to a predictor variable, in our case a SNP genotype. Variables in *U* are unassociated with the SNP, variables in *D* are directly associated with the SNP, and variables in *I* are indirectly associated with the SNP. Indirect association with the SNP implies that an outcome variable’s relationship with the SNP genotype is mediated through an intermediate (directly associated) variable. For each possible partition of variables, a Bayes factor is computed, and the model with the highest Bayes factor can be interpreted as the one that best fits the data.

## Results and discussion

Two papers analyzed the GAW19 expression data in the pedigrees alone without using the genotype or phenotype data provided. The first paper investigated the effects of potential covariates on the gene expression levels. The results would provide insights into how to best adjust for external factors in analyzing expression data. To achieve this, Gallagher et al. [19] used linear regression to investigate the effects of age, sex, medication, blood pressure, hypertension, smoking status, and pedigree membership on the principal components of gene expression levels in this sample. Most of the covariates tested were not significantly associated with the principal components that were generated. However, there was a highly significant correlation between pedigrees and the second principal component, indicating that it is essential to correct expression levels for pedigree membership when the study sample is composed of related individuals. This finding was reflected throughout the analyses reviewed here, as the second paper [20] presents and evaluates an analytic method that directly incorporates pedigree membership into the analysis and most of the subsequent papers employ software that accounts for pedigree membership. Gallagher et al. [19] also investigated the familiality of the proportions of the cell types in the expression samples. To achieve this, information from the Haem Atlas and the Cell Mix software was used to predict the proportions of different types of cells (granulocytes, natural killer cells, monocytes, and B, Tc, and Th lymphocytes) within individuals. Analysis of variance indicated that the proportions of cell types differed significantly among pedigrees, a factor not considered by most investigators.

The second paper [20] reports the development and evaluation of a statistical approach to incorporate family relationships into the estimates of correlations among gene expression levels. Their method is based on WGCNA. Tissier et al. [20] build a coexpression network for each pedigree and summarize the networks for all pedigrees. The results are then compared with WGCNA on the whole set of pedigrees without accounting for pedigree structures as well as with using WGCNA on the residuals of a linear mixed model so as to remove within-family correlation structures. Their work is limited to the 5 largest pedigrees of sizes: 65, 55, 45, 62, and 49. From all modules of these 5 pedigrees, the first significant eigengene was found using the model *Y*_*j*_ = *μ* + *u*_*j*_ + *βeigengene*_*j*_^*k*^ + ɛ_*j*_, where *Y*_*j*_ is the outcome, *u*_*j*_ the random genetic effect, and *eigengene*_*j*_^*k*^ the value of the eigengene of module *k* of family member *j.* The individual genes were identified by taking the intersection and union of the genes in the 5 modules flagged by the 5 most significant eigengenes. The first principal component was computed for each set, and the principal component that explained the largest amount of variance was selected as the eigengene of their approach. Unfortunately, this family based approach did not yield a significant finding under the simulation model used for SBP. However, they were able to identify clusters of genes correlated with the top genes used to simulate SBP. The authors concluded that a thorough simulation study would be required to investigate the method in a more comprehensive way.

The second set of papers investigated 3 aspects of gene expression that query complexity and incorporate functionality to better understand the nature of gene regulation. The papers are summarized in Table 1. The first paper by Cantor et al. [21] uses a linear mixed model statistical approach to investigate the presence of allelic heterogeneity at eQTL. The initial eQTL were chromosomal regions likely to harbor regulatory elements contributing to variation in gene expression. They are identified through linkage analysis of the quantitative expression levels within the pedigrees. Once a region is found to be linked to the expression levels of a particular gene through linkage, individual SNPs within the region are tested for association with the expression levels. Given the current focus on an initial association study, rather than linkage analysis followed by an association study, eQTL have come to refer to the SNPs associated with variation in gene expression. We use the term interchangeably here, depending upon the analysis used to generate the result.

**Table 1:** Contribution of the “Genetics of Gene Expression” subgroup

			Real data				
Paper	Sample size	Relatedness correction	Phenotype	Genotype	Simulated data	Analytic approach	Results
Cantor [21]	653 R	SOLAR-MGA and FaST-LMM for expression and simulated traits	TIMM10 and LR8 expression probes	6 MAP4 SNPs 47 and 180 in TIMM10, LR8	200 replicates of SBP. DBP and Q1	Type 1 error and power estimated. Single SNPs and Sequential conditioning with SOLAR-MGA and FaST-LMM	Software results similar. Multiple independent SNPs associated with eQTL, supporting complexity
Howey [23]	954 R	GEMMA for expression, FaST-LMM for BP	11 expression probes, SBP DBP, HTN, PP and MAP	44 candidate HTN SNPs 14 SNP-SNP inter-actions	Not used	FaST-LMM for HTN related phenotypes, GEMMA for SNP–SNP interactions. Linear regression using PLINK	SNPs not significant. 2 SNP–SNP interactions with expression
	1946 U						
Peralta [22]	959 R	variance components within SOLAR	20527 gene expression values	10552 potential allele specific DNase hypersensitivity sites	Simulated 10,000 heritable quantitative phenotypes	Covariance kernels (weighted and nonweighted for DHS likelihood) using 10,552 SNPs, as predictors of gene expression	10 transcripts associated with weighted DHS kernel, 8 associated with nonweighted kernel

*DBP* diastolic blood pressure; *DHS* DNase hypersensitivity site; *eQTL* expression quantitative trait locus; *HTN* hypertension; *MAP* mean arterial pressure; *PP* pulse pressure; *R* related individuals; *SBP* systolic blood pressure; *SNP* single nucleotide polymorphism; *SNV* single nucleotide variant; *U* unrelated individuals

Cantor et al. [21] used the SOLAR-MGA [18] and FaST-LMM [28] software to account for the nonindependence of pedigree members in the GAW19 expression data. Prior to investigating the real data, type 1 statistical errors and power were estimated using the simulated trait data provided for workshop participants. This group reports comparable power and type 1 errors for the two programs for analyses examining individual SNPs. However, the investigation of eQTL allelic heterogeneity is based on analyses that sequentially condition the likelihood of the data on significant SNPs until no additional individual SNP associations are observed. Again simulated data show the programs are comparable.

In the second paper listed in Table 1, Howey et al. [23] were able to find that not only is there allelic heterogeneity in expression levels, but that there is also evidence that pairs of alleles interact significantly to contribute to these traits. The authors explored 48 published SNP associations with blood pressure and expression traits in the GAW19 sample. Similar to Cantor et al. [21], family relationships were taken into account using linear mixed models as programmed in FaST-LMM. Marginal SNP associations with blood pressure–related traits were not significant; 13 SNP interaction pairs, however, were tested using the GEMMA (genome-wide efficient mixed-model analysis) [30] software, which uses an estimated kinship matrix, and the authors report evidence of interactions contributing to expression levels. Specifically, there were 2 clear SNP × SNP interactions, as well as an overall *p* value of 0.017 for simultaneously testing all considered interactions, supporting this aspect of eQTL complexity along with allelic heterogeneity.

In the third paper listed in Table 1, Peralta et al. [22] present an approach to detect *cis*-regulatory loci using the physical property of allele-specific chromatin accessibility as weights in a kernel variance component test. *Cis* loci are those within a small distance of the gene on the chromosome and would represent local gene regulation. Their work is based on the observation that chromatin remodeling processes, such as those associated with transcription, create openings in the chromatin that can be detected as DNase-I hypersensitivity sites. They allow transcription factors to interact with the underlying DNA. The functional potential of a locus was estimated as the departure from the expected equal chromatin accessibility of 2 alleles within a locus.

The authors constructed 2 covariance kernels from the GAW19 data. One was weighted by the functional potential of the SNPs, specifically based on those known to be heterozygous at DNase-I hypersensitivity sites in the public ENCODE (Encyclopedia of DNA Elements) genome DNase-I sequence data. These covariance kernels were then used in a test of association. The authors used a variance component approach with both the weighted and nonweighted covariance kernels to test genetic variants with putative allele specific effects on gene regulation. The SOLAR software was used construct these association tests in the presence of the pedigree structures. Evidence of potential *cis*-regulatory effects for 10 transcripts was detected, 2 of which appeared only when the weightings were used in the analysis. One may conclude from this work that incorporating evidence of functionality can improve the detection of *cis-*regulatory elements.

The third set of papers, summarized in Table 2, investigated the relationship between gene expression and phenotype (blood pressure or hypertension), with or without making use of genetic data. Most papers focused on pairwise relationships between variables, testing separately for association between genotype and gene expression and/or between gene expression and phenotype, but Tong et al. [26] used a similarity-based weighted U statistic approach to jointly model effects of genotype and gene expression on phenotypes, and found some advantage (in terms of significance levels of association analyses) by incorporating both genotype and gene expression measures into the analyses. Ainsworth and Cordell [27] took forward individual gene expression probes and WGCNA module eigengenes showing association with phenotype (SBP and DBP), together with SNPs that associated with the relevant gene expression measures, for causal modeling using either SEM or a BUF [29]. Although only weak overall levels of significance were observed, SEM and BUF were generally in agreement in their assessment of the underlying causal model, with the most commonly identified model suggesting a causal pathway that led from SNP genotype through gene expression to phenotype. Pitsillides et al. [24] identified the same associations between gene expression and blood pressure that had been identified by Ainsworth and Cordell [27], and also identified many highly significant associations between genotype and gene expression of nearby genes (so-called *cis*-eQTL), with a significant enrichment of *cis*-eQTL occurring in known blood pressure loci and regulatory regions. Radkowski and Wątor [25] identified gene expression probes in 6 well-annotated genes that are potentially predictive of hypertension dynamics (change in blood pressure over time). Although the overall predictive accuracy was not great (cross-validation R^2^ of 0.1459), 2 of the genes identified, *IFNAR1* and *NOX3*, have previously been implicated in the pathogenesis of hypertension.

**Table 2:** Contributions of the “Genetics of Gene Expression and Phenotype” subgroup

			Real data				
First author	Sample size	Relatedness correction	Phenotype	Genotype	Simulated data	Analytic approach	Results
Ainsworth [27]	R 638	GWAS: FaST-LMM	Covariate adjusted mean SBP, DBP	427,952 GWAS SNPs	Analyzed but not presented	Pairwise association, WGCNA to identify variables for causal modeling in SEM and BUF	Weak significance, high concordance between SEM and BUF
		WGCNA SEM, BUF: none					
Pitsillides [24]	R 267	Linear mixed effects models	DBP, SBP	12,296,048 SNVs from WGS	Not used	Test of enrichment of *cis*-eQTL in known BP loci and regulatory regions; pairwise association of expression and BP	Many highly significant eQTL. Enrichment of eQTL in known BP loci and regulatory regions
Tong [26]	U 142	None needed	SBP, DBP, HTN, adjusted for covariates	6,956,910 SNVs from WGS in 17,558 genes	Not used	Similarity-based test for joint effects of genotype, gene expression, phenotype	Weak significance, but some benefit from using genotypes and gene expression
Radkowski [25]	R 340	None made	HTN at several time points; change in BP	Not used	Not used	Change in BP (in individuals with no HTN) modeled as a function of gene expression and covariates	7 potentially predictive HTN gene expression probes identified in 6 genes

*BP* blood pressure; *BUF* Bayesian unified framework; *DBP* diastolic blood pressure; *eQTL* expression quantitative trait locus; *GWAS* genome-wide association study; *HTN* hypertension; *R* related individuals; *SBP* systolic blood pressure; *SEM* structural equation modeling; *SNP* single nucleotide polymorphism; *SNV* single nucleotide variant; *U* unrelated individuals; *WGCNA* weighted gene correlation network analysis; *WGS* whole-genome sequencing

## Conclusions

The GAW19 gene expression group used the expression, genotype, and phenotype data (both real and simulated) provided by the organizers to apply, develop and evaluate analytic methods and pursue biological questions relevant to an overarching goal of understanding the regulation of gene expression data in genetically complex disorders. Most contributions capitalized on or corrected for the fact that these data were measured on multiple members of large pedigrees, and thus the data could not be treated as study samples of independent individuals. Most frequently used analytic methods for expression data are based on this assumption of independence of members of the study sample and most studies are conducted on samples of unrelated individuals. Thus, analysis of the pedigree data was a challenge and an opportunity for the members of our gene expression group. The family structures allowed Gallaugher et al. [19] to investigate familiality of gene expression, Tissier et al. [20] to develop a method to incorporate family structures in the WGCNA gene expression studies, Cantor et al. [21] to apply and compare linear mixed models approaches that have recently been developed, and Howey et al. [23] to incorporate linear mixed models into the testing of gene interactions for the expression traits.

To investigate familiality of gene expression, Gallaugher et al. [19] conducted a principal components analysis of the gene expression data, and tested the principal components for correlations with several potential covariates, where one of them was pedigree membership. The strongest covariate association was a highly significant association across the pedigrees for the second principal component and average gene expression. Three pedigrees were considered outliers. The group reasoned that this phenomenon could be due either to pedigree-specific genetic variation, technical artifacts, or clinical factors. Technical artifacts were ruled out, however, regardless of its cause, Gallaugher et al. [19] concluded that such familiality should be addressed when pedigree members are included in an analysis so as to avoid type 1 statistical errors. In addition, these investigators added to the growing body of evidence supporting the notion that gene expression is familial. Identifying the factors contributing to this familiality, such as tissue type or stage of development, will lead to a better biological understanding of the process and result in improved study designs.

In complementary work, Tissier et al. [20] developed their own method to incorporate family structure into the gene expression studies using WGCNA. This is a valuable addition to a method designed for the analysis of independent individuals. The authors built a coexpression network for each family and then combined the results across families. Comparison was conducted with 2 simpler approaches: (a) ignoring correlations among the family members and (b) decorrelating the gene expression by using the residuals of a mixed model and a single-probe analysis. That is, with the lack of simulated gene expression data, they had to evaluate their approach by contrasting their results with those of the other methods. Although, not definitive, they found that their method provided more significant results than the other two. Additional research on their approach could provide an important addition to WGCNA.

To address pedigree structure with existing software, Cantor et al. [21] used linear mixed models to identify eQTLs with SOLAR-MGA and FaST-LMM, and Howey et al. [23] used FaST-LMM and GEMMA (genome-wide efficient mixed-model analysis). Unlike Tissiers et al’s [20] approach, this software is designed for a simple assessment of SNP association and interactions and to identify *cis*-eQTL. Cantor et al. [21] found that for both single SNP assessment and conditional analyses, these programs had similar type 1 and type 2 errors, and the type 1 errors were as expected for both programs. The advantage of FaST-LMM is that kinship relationships among the individuals do not have to be made explicit. The pedigree data with simulated phenotypes provided by the Workshop allowed an evaluation of this software for related individuals, and thus these programs are recommended to investigators exploring expression data with pedigrees.

Three papers focused on biological aspects of gene expression, Peralta et al. [22] used biological complexity to develop a statistically rigorous method to incorporate a biological feature of genes, DNase-I hypersensitivity sites, into tests of association. They built 2 covariance kernels, one nonweighted and one weighted by the functional potentials, and conducted kernel-based variance component association analysis. They found evidence of potential cis regulatory effects, where a stepwise removal of the *cis*-located SNPs from the weighted kernel results in a nonsignificant association signal. Using this methodology, they found evidence of allele-specific *cis*-regulation for 4 transcripts with both kernels.

Addressing the potential complexity of *e*QTL, Cantor et al. [21] provided evidence of allelic heterogeneity at *cis*-eQTL, and Howey et al. [23] illustrated a second source of complexity by testing for and identifying epistasis in gene regulation. We thus have evidence of (at least) 2 sources of complexity in the genetics of gene expression.

The papers in our group that incorporated genotype and phenotype data together with measurements of gene expression investigated whether the integration of these different data types (either, explicitly through joint modeling, or implicitly, through incorporation of prior biological knowledge) could generate improved levels of statistical significance and/or increased biological understanding regarding underlying causal mechanisms. Although only weak overall levels of significance were observed with regard to phenotype, in general the incorporation of different data types was found to be somewhat advantageous in terms of generating improved levels of significance and providing more interpretable biological models.
